# Mortality Records Guide UAV-Based Assessment of Power-Line Conspicuity at a Black-Necked Crane Breeding Site

**DOI:** 10.3390/ani16172698

**Published:** 2026-08-31

**Authors:** Xintong Li, Zhengwu Pan, Yumin Guo

**Affiliations:** School of Ecology and Nature Conservation/School of National Park, Beijing Forestry University, Beijing 100083, China; lxt940706@163.com (X.L.);

**Keywords:** Black-necked Crane, power-line collision, image conspicuity, unmanned aerial vehicle, conservation monitoring, mitigation prioritization

## Abstract

Overhead power lines increasingly intersect alpine wetland and grassland habitats on the Qinghai–Tibet Plateau, but variation in wire conspicuity within collision-associated areas remains poorly quantified. We combined GPS-GSM tracking and field verification to document infrastructure-associated mortality and separately used standardized UAV RGB images to characterize power-line conspicuity at the Luanhaizi Wetland, Qinghai Province. From 2020 to November 2025, we monitored 40 Black-necked Cranes (Grus nigricollis); nine were alive at the data cutoff, 23 were confirmed dead, and eight were lost to follow-up. Among the confirmed deaths, nine were classified from field evidence as wire-mesh fence entanglements and 14 as power-line collisions. All 14 field-inferred collision deaths involved 110 kV lines in this monitored sample. Accordingly, the UAV assessment focused on a selected section within the documented 110 kV collision-associated area; this site-selection criterion does not demonstrate that 110 kV lines are intrinsically more hazardous than other local voltage classes. We analyzed 640 UAV images and calculated an image-based visibility index (VI) combining normalized RGB target–background contrast and edge strength. VI describes relative wire conspicuity in standardized photographs rather than Black-necked Crane visual perception or collision probability. Blocked non-parametric tests showed that VI was lower under overcast than clear conditions (*p* < 0.001) and varied with observation distance and relative UAV height (both *p* < 0.001), whereas viewing angle was not significant (*p* = 0.065) and time of day showed a weak effect (*p* = 0.030). VI rankings remained reasonably stable under alternative component weightings. This two-stage approach uses documented mortality to identify candidate sections for image-based assessment but does not test whether low VI predicts collisions. It can support targeted marker trials that require independent evaluation through standardized collision monitoring.

## Simple Summary

Power lines and livestock fences can injure or kill large birds, but managers often do not know which structures should be examined first. We monitored 40 Black-necked Cranes at a breeding site in Qinghai, China, from 2020 to 2025 and combined tracking records with field searches and standardized drone photographs. Of 23 confirmed deaths, nine were linked to wire-mesh fence entanglement and 14 to overhead power-line collisions. All 14 field-inferred collision deaths involved 110 kV lines in this monitored sample; this does not demonstrate that 110 kV lines are intrinsically more hazardous than other voltage classes. We therefore analyzed 640 RGB photographs acquired from a selected section within the documented 110 kV collision-associated area to characterize how the image-based conspicuity of the wires varied among acquisition conditions. Conspicuity was generally lower under overcast skies and also varied with observation distance and relative drone height. The resulting index describes wire appearance in RGB photographs and does not reproduce crane vision or estimate collision probability. Mortality records can identify candidate sections for detailed photographic assessment, after which the effectiveness of highly visible markers should be tested through controlled field monitoring. Fence removal or modification represents a parallel recommendation based on the documented mortality records.

## 1. Introduction

Overhead power-line networks increasingly overlap with bird habitats, including wetlands and grasslands in ecologically sensitive regions [1,2]. This is particularly relevant on the Qinghai–Tibet Plateau, where transmission infrastructure has expanded across areas used by large-bodied waterbirds [3,4]. Collisions with conductors and shield wires are a recognized source of mortality, but their occurrence varies with line configuration, landscape setting, and flight behaviour [5,6,7]. Overhead power lines cause bird mortality primarily through collision and electrocution. Collision is especially relevant to large-bodied species because conductors and shield wires may not be detected early enough for avoidance [6,8]. Reports of bird–wire collisions date to the nineteenth century, including a mass-mortality event documented in 1876 [9]. As transmission and distribution networks expand, such collisions remain a persistent conservation concern.

Wire conspicuity can vary with illumination, weather, background, and viewing geometry [10,11,12], but the visual resolution of a conductor is only one component of collision risk. Collision probability also depends on flight trajectory, altitude and speed, head and body orientation, avoidance behaviour, line configuration, and the ecological context in which the line is encountered [7,12,13]. Studies of avian visual fields show that head position can create substantial blind regions in the direction of travel in some large birds [13,14]. Consequently, a static RGB image acquired from a UAV cannot reproduce the retinal view or avoidance decisions of a flying crane. Reduced target–background contrast in such an image should therefore be interpreted as lower image-based conspicuity, rather than as evidence of a higher collision probability. Whether lower photographic conspicuity corresponds to delayed detection or increased collision occurrence remains a hypothesis requiring behavioural or field validation.

Collision mitigation includes routing new lines away from important bird habitats [15], undergrounding, and increasing wire conspicuity with visual markers [16,17]. Undergrounding removes the aerial obstacle but is often impractical across extensive existing networks because of engineering and economic constraints [18]. Visual markers are therefore widely used, although their effectiveness varies among species, landscapes, environmental conditions, and designs [16,17]. Post-installation monitoring is needed to determine site-specific outcomes [19,20]. For unmarked lines, field measurements can characterize how wire conspicuity varies among standardized acquisition conditions.

The Black-necked Crane (*Grus nigricollis*) is listed as a Class I nationally protected species in China and is the only crane species that lives year-round on high-altitude plateaus [21]. Its global population was estimated at 17,500 individuals in 2023 [22]. On the Qinghai–Tibet Plateau, it breeds in open wetland–grassland mosaics, where power-grid infrastructure continues to expand [3,23,24,25]. Family groups move between nesting and foraging areas, and overhead lines crossing these routes may pose collision hazards [6,26,27,28]. Although habitat protection and artificial nesting platforms have improved breeding outcomes at some sites, habitat disturbance and human-made infrastructure remain conservation concerns [29,30,31].

Research on the Black-necked Crane has focused mainly on migration routes [32], activity rhythms [33], foraging behaviour [34], and nest-site selection [35], whereas infrastructure-related mortality at breeding sites has received less attention. Fence entanglements and power-line collisions have nevertheless been reported [31]. Site-based collision assessments commonly use mortality records or infrastructure characteristics to identify conflict areas [36], but mortality records alone do not quantify flight exposure or collision probability. Movement-based approaches provide a complementary and more direct means of spatial risk estimation by integrating GPS trajectories, flight altitude, and power-line geometry. For example, Pay et al. [37] modelled the probability that tracked birds would cross power lines at hazardous altitudes and evaluated the resulting spatial risk predictions against independent fatality locations. By contrast, the present study uses field-inferred mortality records to select a collision-associated power-line section and then characterizes variation in the image-based conspicuity of that sampled line under standardized UAV acquisition conditions. Target–background contrast and edge definition can describe relative wire conspicuity in RGB photographs, but they do not reproduce Black-necked Crane visual perception or independently establish collision risk [13,38].

Accordingly, this study aimed to: (1) summarize monitoring outcomes and recorded mortality causes; (2) map power-line collision mortalities and define a collision-associated 110 kV UAV sampling area; (3) construct an image-based visibility index (VI) from contrast and edge strength; and (4) apply VI to quantify relative power-line conspicuity under standardized UAV acquisition conditions, not collision risk.

## 2. Materials and Methods

### 2.1. Study Area

**Figure 1:**
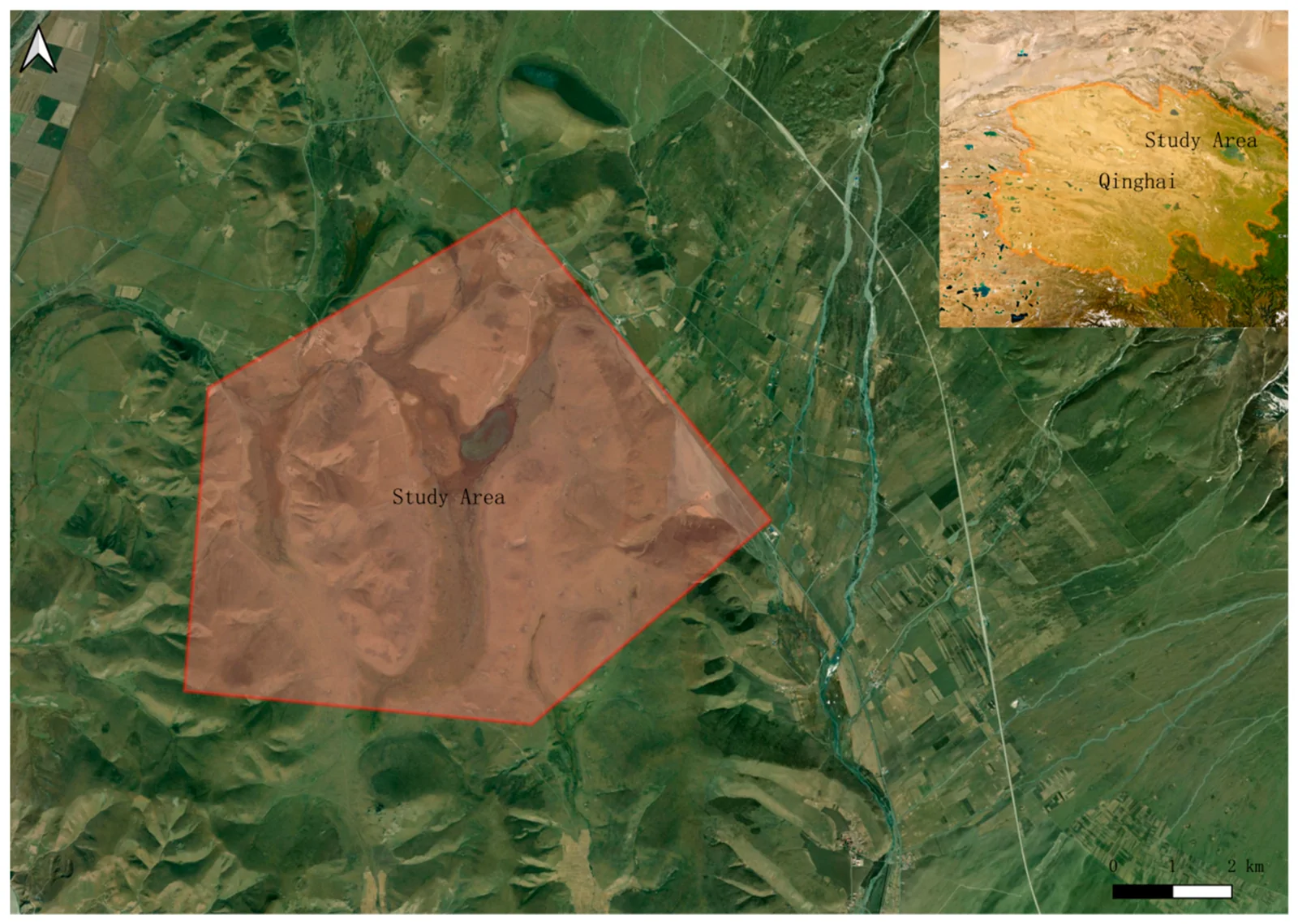
Geographic location of the work site: Luanhaizi Wetland, Menyuan County, Qinghai Province, situated on the southern slopes of the Qilian Mountains, northeastern Qinghai–Tibet Plateau. Basemap: Esri World Imagery.

This study was conducted in Luanhaizi Wetland, Menyuan County, Qinghai Province, China (approximately 37.48–37.75° N, 101.20–101.38° E; Figure 1), on the southern slopes of the Qilian Mountains in the northeastern Qinghai–Tibet Plateau. The area comprises an alpine wetland and grassland mosaic and supports multiple breeding family groups of Black-necked Cranes each year. The local overhead power-line network includes two 110 kV lines, one 35 kV line, and several 10 kV and 0.4 kV distribution lines, some of which cross areas used by cranes for breeding and foraging. Livestock wire-mesh fences also occur within parts of the wetland and surrounding grassland used by crane families. Together, these infrastructures provided the field setting for summarizing infrastructure-related mortality and for using collision records to select a line for UAV assessment.

### 2.2. Capture, Banding, GPS-GSM Tracking, and Field Monitoring

From 2020 to 2025, we monitored 40 Black-necked Cranes at Luanhaizi Wetland. Thirty-eight young cranes from four breeding families and one adult not assigned to a family were captured, banded, and fitted with GPS-GSM trackers. The untagged adult male of Family 2 (GS) was monitored through repeated field observations. This bird was included in the descriptive summary of monitoring outcomes and mortality but excluded from analyses requiring GPS locations or time since tracker deployment. Captures took place between June and August. Young cranes were captured before they could sustain flight, after their body mass exceeded 2 kg and while they still responded to approach by crouching in wetland vegetation.

Experienced field workers first located each bird from elevated ground using spotting scopes. They then approached on foot and captured the bird with a hand net. Each captured bird was restrained with a handling cloth, weighed, fitted with a uniquely coded red leg band, and equipped with an HQLG4037S leg-mounted GPS-GSM tracker (37–44 g; Hunan Global Messenger Technology Co., Ltd., Changsha, China). The trackers recorded GPS locations and transmitted the data through the GSM network. Each GPS–GSM tracker weighed less than 3% of the corresponding bird’s body mass, following the conservative device-load guideline commonly applied in avian telemetry [39]. Handling usually lasted less than 10 min, after which each bird was released at its capture location. The red leg bands were supplied by the National Bird Banding Center of China. All capture, handling, colour-ringing, and transmitter-deployment procedures were conducted under administrative permits issued by the National Forestry and Grassland Administration of China for the relevant field seasons and followed the animal-welfare safeguards described in the Institutional Review Board Statement.

We reviewed incoming locations through the tracking platform. Prolonged immobility, abrupt cessation of movement, a marked decline in activity, or convergence between tracker-recorded and ambient temperatures prompted a field check. Field staff or local volunteers then searched the last recorded location and its surroundings.

Mortality causes were classified using concordant GPS-GSM and on-site evidence. All 14 power-line collision cases were investigated in the field, and none was assigned from signal loss alone. Evidence included terminal abnormal or stationary locations beneath or immediately adjacent to a power line and collision-compatible external trauma, such as visible injuries or fractures of the wings, legs, or neck. Where external trauma could not be documented, classification required concordant terminal tracking and field-recovery evidence directly beneath the line. Fence-related mortality required direct evidence of entanglement. Because standardized necropsy was unavailable, these classifications are conservatively termed field-inferred rather than necropsy-confirmed.

At the November 2025 cutoff, the eight lost birds had permanent signal loss without field evidence of mortality, recorded intersections with overhead power lines, or terminal clustering near lines; they were censored at their last valid record and excluded from confirmed-death percentages. For tagged birds, follow-up extended from deployment to confirmed death, loss, or cutoff; mortality incidence per bird-year was reported with exact Poisson 95% confidence intervals, and GS was excluded from person-time analyses. Sensitivity analyses assigned 0, 2, 4, 6, or 8 unobserved deaths among lost birds and recalculated total mortality and the proportional contribution of the 14 observed power-line deaths.

To illustrate family-level spatial distributions during the early pre-flight and later flight-capable sampling periods, we used GPS-GSM locations collected in 2023. The early period extended from tracker deployment to 1 September, and the later period from 7 to 20 October. Locations were grouped by family and plotted as individual points in QGIS. The resulting maps provide descriptive representations of the recorded locations rather than formal estimates of space-use extent.

### 2.3. UAV-Based Monitoring Approach

UAV imagery enables standardized photographs to be collected from predefined viewing positions [40,41,42,43]. Using the GPS-GSM and field-verification records described in Section 2.2, we selected one unmarked span within the documented 110 kV collision-associated area. RGB photographs were acquired using a DJI Phantom 4 UAV (DJI, Shenzhen, China). Holding the underlying line configuration and general spatial setting constant allowed environmental and acquisition effects on image-based line conspicuity to be evaluated under controlled field conditions.

Image acquisition followed a factorial design with six factors: observation distance from the line (20, 50, 80, and 100 m); UAV height above ground level (11, 16, 20, and 25 m), which positioned the camera near the lowest conductor, middle conductor, shield wire, and above the shield wire, respectively; time of day (morning, shortly after sunrise; evening, shortly before sunset); weather condition (clear and overcast); viewing direction (eastward and westward); and relative horizontal viewing angle (30°, 60°, 90°, 120°, and 150°). The two viewing directions represented image acquisition from opposite sides of the line. When combined with the five relative viewing angles, they corresponded to ten absolute camera headings (30°, 60°, 90°, 120°, 150°, 210°, 240°, 270°, 300°, and 330°). The four height levels described UAV camera positions relative to the line structure and were not treated as measured crane flight heights.

The design comprised 4 × 4 × 2 × 2 × 2 × 5 = 640 experimental combinations. Spatially, these combinations represented one line span and 160 nominal camera-geometry positions (4 distances × 4 heights × 2 viewing directions × 5 viewing angles), each sampled under the four time-of-day × weather combinations. Five photographs were captured per combination, yielding 3200 photographs, all meeting the minimum technical-quality requirements. The photograph with the clearest focus, most appropriate exposure, and least target obstruction was retained from each combination. Selection was quality-based rather than random; the other four photographs served as technical quality-control repeats and were not independent observations. The final dataset contained 640 photographs, one per combination, obtained from the same span with the same UAV–camera system. They provided intensive within-span coverage of the predefined conditions and were treated as factorial rather than spatial replicates. UAV positions standardized acquisition geometry rather than crane flight trajectories. The design provides a standardized image-assessment protocol applicable in other power-line settings and landscape contexts.

### 2.4. Analytical Methods

All 640 images retained after the quality screening described in Section 2.3 were analyzed at a resolution of 4000 × 2250 pixels. Visible phase conductors and shield wires were manually traced as polylines using the VGG Image Annotator (VIA, Oxford, UK, version 2.0.12) [44]. VIA served only as an annotation interface; no automated segmentation or deep learning model was used. The annotation coordinates were exported and converted into binary line masks. Visible length was calculated as the summed Euclidean distance, in pixels, between consecutive vertices of all annotated polylines in each image. Conductor diameter was not included as a separate explanatory variable.

To examine annotation repeatability, 63 images, representing 9.8% of the final dataset, were annotated a second time using the same procedure. These images covered a range of acquisition conditions in the experimental design. The first and second annotations were converted into binary masks and compared using precision, recall, F1 score, and intersection over union (IoU). Overall pixel accuracy was not used because power-line pixels occupied only a small proportion of each image, and the large number of background pixels would have inflated this measure.

For each image, the local background was defined by applying a 100-pixel dilation to the power-line mask and then removing the annotated line pixels from the dilated region. This adjacent region was used instead of the entire image background because distant areas of vegetation, water, or sky did not necessarily form the immediate visual background of the wire. Target–background contrast was calculated as the Euclidean distance between the mean camera-encoded RGB vectors of the annotated line pixels and the local background pixels:

$$\mathrm{Contrast}\;=\;\left\|\bar{I_{\mathrm{line}}}-\bar{I_{\mathrm{bg}}}\right\|$$

where $\bar{I_{\mathrm{line}}}$ and $\bar{I_{\mathrm{bg}}}$ are the mean camera-encoded RGB values of annotated power-line and adjacent-background pixels, respectively. Larger values indicate stronger numerical separation between these vectors. Because RGB is device-dependent and perceptually non-uniform, contrast was treated as an image descriptor rather than a perceptual colour difference for Black-necked Cranes.

Image sharpness was assessed using the variance of the Laplacian operator, a commonly used focus measure in image processing, while edge strength within the power-line regions was quantified using Sobel gradient magnitude and Canny edge detection [45,46]. In this workflow, the Laplacian operator was used to describe overall image focus, whereas the Sobel gradient and Canny edge detection were used to characterize local edge transitions and thin edge structures along the annotated power-line regions. The original image was first converted to grayscale, and the Laplacian operator was applied to compute the overall edge response. The variance of this response was used as the sharpness metric. To quantify edge sharpness specifically around power lines, the Sobel operator was used to compute the image gradient magnitude. The gradient values were summed within the regions where the power-line mask equaled one, yielding the cumulative edge strength value (E). In this context, sharpness characterizes the overall clarity of the image, while edge strength focuses on the concentration of edge sharpness along the power-line segments. After contrast and edge strength were obtained for each image, both metrics were rescaled using min–max normalization, a standard preprocessing step for placing variables with different numerical ranges on a common scale [47], based on the observed range in the final 640-image dataset. For contrast, the observed minimum and maximum were 0.097538088 and 48.50062026, respectively. For edge strength, the observed minimum and maximum were 427,208.1179 and 87,732,949.65, respectively. The normalized components were calculated as follows:

Contrast_norm = (Contrast − 0.097538088)/(48.50062026 − 0.097538088) Edge_norm = (Edge_strength − 427208.1179)/(87732949.65 − 427208.1179)

The visibility index (VI) was then calculated as the arithmetic mean of the two normalized components and multiplied by 100 for easier interpretation:

VI = 100 ∗ (Contrast_norm + Edge_norm)/2

Equal weighting was used as a transparent baseline summary because contrast and edge strength describe complementary image features: target–background separation and local structural definition. The 0.5/0.5 formulation was not treated as a species-specific perceptual weight or calibrated detectability function. To examine numerical dependence on this choice, alternative VI values were calculated as VIw = 100 × [w × Contrast_norm + (1 − w) × Edge_norm], where w was 0.25, 0.50, or 0.75. Rank consistency with the equal-weight VI was evaluated using Kendall’s τ and Spearman’s ρ. We also re-applied the one-dimensional K-means stratification and compared the resulting High-concern groups using Jaccard similarity. This analysis assessed robustness of rankings and class membership to weighting, not biological validity. Contrast and edge strength were retained separately at image level, while VI served as a compact composite for comparing standardized RGB photographs. Visible length remained supplementary.

Contrast, image sharpness, edge strength, visible length, VI, and the associated image metadata were stored in a CSV file. The 640 images were obtained from a structured factorial design, and observations that differed only in the level of the focal factor formed matched sets rather than independent replicates. Each experimental factor was therefore analyzed using a blocked non-parametric test, with every unique combination of the other five factors treated as a block. The two-level factors, comprising weather condition, time of day, and viewing direction, each produced 320 matched pairs and were analyzed using two-sided Wilcoxon signed-rank tests. Observation distance and UAV height each produced 160 blocks containing four levels, whereas relative viewing angle produced 128 blocks containing five levels; these factors were analyzed using two-sided Friedman tests. VI was the primary response variable, while visible length was examined as a supplementary measure. The block structure preserved factorial pairing, while the single physical span, UAV platform, and camera sensor were held constant. The 640 observations therefore represent factorial image-acquisition combinations within this controlled setting rather than spatially replicated spans. Line span was not included as an additional blocking factor because it had only one level. Statistical significance was assessed at α = 0.05. *p*-values were reported to three decimal places when *p* ≥ 0.001 and as *p* < 0.001 when smaller.

One-dimensional K-means was used as an algorithmic aid to produce a descriptive summary of the VI distribution, rather than to infer naturally distinct ecological clusters [48]. Candidate solutions with k = 2–6 were compared using the within-cluster sum of squares (WCSS) and mean silhouette score [49]. Although k = 2 had the highest silhouette score, k = 3 was intentionally retained to preserve an intermediate screening category. K-means was run with k-means++ initialization, a maximum of 300 iterations, and 50 restarts. The resulting groups were ordered by centroid. Because lower VI indicated lower image-based line conspicuity, the groups with low, intermediate, and high VI centroids were labelled High concern, Medium concern, and Low concern, respectively. Cut-points were defined as the midpoints between adjacent centroids. These data-derived classes summarize relative image variation within the present dataset and do not represent universal conspicuity or collision-risk thresholds.

For the 63 images annotated twice, annotation variability was further propagated through the complete metric-calculation workflow. Target–background contrast, edge strength, and VI were recalculated separately from the first and second annotations. Both sets of measurements were normalized using the fixed minimum and maximum values obtained from the complete 640-image dataset, rather than normalization ranges recalculated from the repeatability subset. Agreement between the two VI estimates was evaluated using Pearson’s correlation coefficient, Spearman’s rank correlation coefficient, Kendall’s τ, the single-measure absolute-agreement intraclass correlation coefficient [ICC(A,1)], mean absolute error (MAE), median absolute difference, root mean square error (RMSE), mean bias, and 95% limits of agreement. The ICC variance components were also used to partition total VI variance into between-image, annotation-round, and residual components. A paired Wilcoxon signed-rank test was used to examine whether VI differed systematically between annotation rounds. Both sets of VI values were also assigned to the existing three concern classes using the class boundaries derived from the original 640-image dataset. Classification repeatability was assessed using exact agreement and Jaccard similarity for the High-concern class.

Robustness to the local-background definition was assessed for all 640 images using elliptical dilation kernels of 50 × 50, 75 × 75, 125 × 125, and 150 × 150 pixels, with the 100 × 100-pixel kernel as the reference. The same annotations, fixed normalization ranges from the original dataset, and unchanged class cut-points were used throughout. Alternatives were compared with the reference using Spearman’s ρ, Kendall’s τ, VI MAE, exact three-class agreement, and High-concern Jaccard similarity.

A shallow classification and regression tree (CART) was subsequently fitted to distinguish High-concern from Not-High observations and to summarize the sampled conditions associated with low image-based line conspicuity [50]. Weather condition, observation distance, UAV height, time of day, viewing direction, and relative viewing angle were included as predictors. All predictors were treated as categorical experimental factors and one-hot encoded before model fitting. This prevented intermediate split values between the sampled distances or heights from being interpreted as continuous ecological thresholds. Tree size was constrained using the Gini criterion, a maximum depth of three, a minimum of ten observations per terminal node, balanced class weights, and minimal cost-complexity pruning. A fixed random seed was used.

Internal classification performance was evaluated using grouped five-fold cross-validation. Groups were defined by the combination of observation distance, UAV height, time of day, viewing direction, and relative viewing angle. Clear and overcast images obtained under otherwise identical acquisition settings were therefore assigned to the same fold. Performance was summarized using precision, recall, and F1 score for the High-concern class, together with overall accuracy. The CART model was used only to identify combinations among the sampled factor levels; it was not used to infer continuous ecological thresholds or predict collision probability.

All analyses were conducted in Python version 3.10. SciPy was used for the Wilcoxon and Friedman tests, scikit-learn [47] for K-means and CART, and pandas and NumPy for data handling and calculations. Fixed random seeds were used where applicable.

## 3. Results

### 3.1. Breeding Status and Mortality Analysis of Black-Necked Cranes

**Figure 2:**
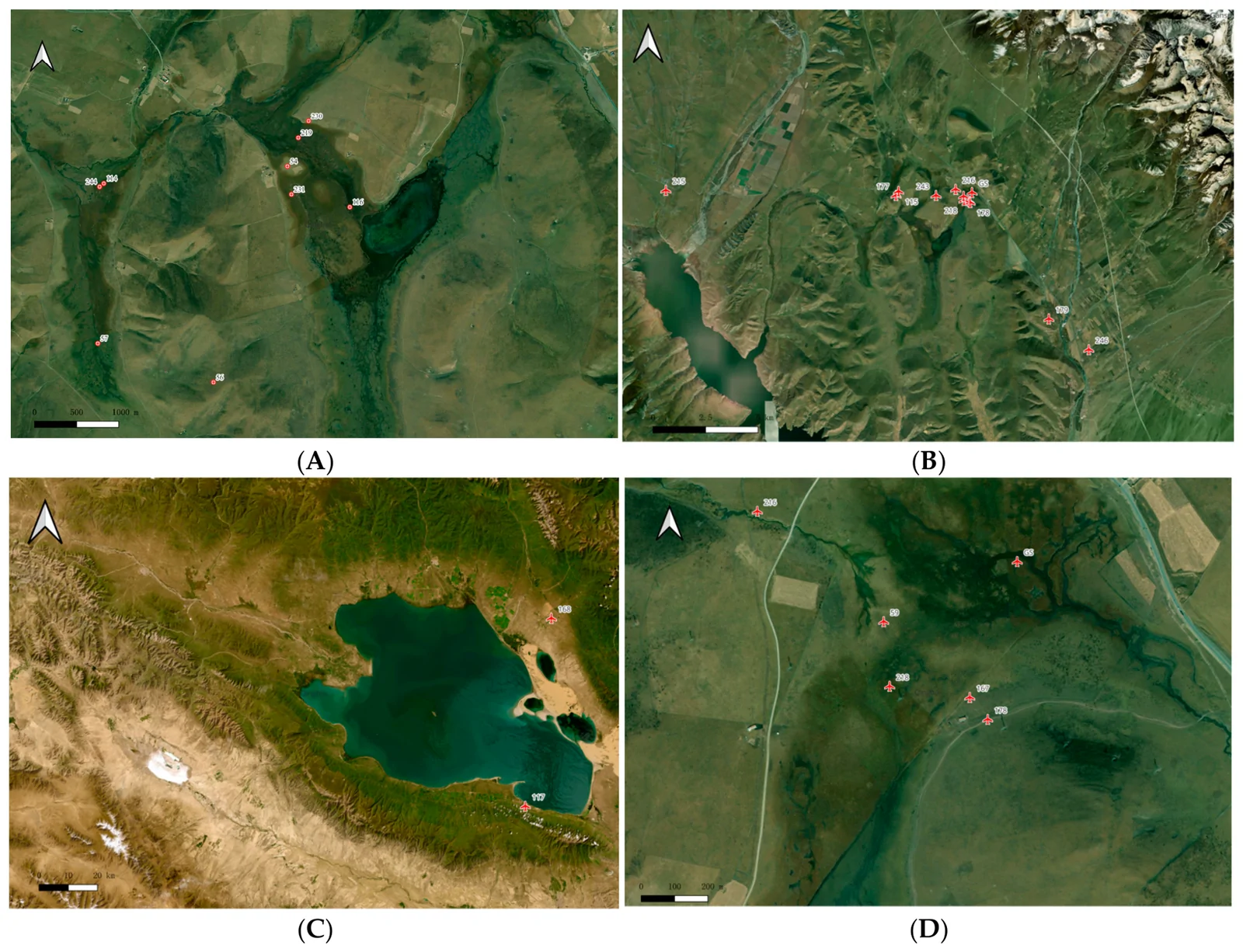
(**A**) Field-inferred fence-entanglement mortality sites in Luanhaizi Wetland; (**B**) field-inferred power-line collision mortality sites in Luanhaizi Wetland; (**C**) field-inferred mortality sites of monitored individuals elsewhere in Qinghai; and (**D**) field-inferred power-line collision locations within the documented 110 kV collision-associated UAV sampling area. Basemap: Esri World Imagery.

From 2020 to 30 November 2025, we monitored 40 Black-necked Cranes at Luanhaizi Wetland: 39 tagged birds and the untagged adult GS. At the cutoff, nine were alive, 23 were confirmed dead, and eight were lost (Table 1). The tagged birds contributed 37.66 bird-years; 22 confirmed deaths yielded 0.584 deaths per bird-year (exact 95% CI, 0.366–0.884), with cause-specific rates reported in Table S5. GS was included in descriptive counts but excluded from person-time estimates. Of the 23 confirmed deaths, nine were field-inferred fence entanglements (Figure 2A) and 14 were field-inferred power-line collisions. Twelve collisions occurred within Luanhaizi Wetland (Figure 2B), and two occurred elsewhere in Qinghai after spring return (Figure 2C). All involved 110 kV lines; no confirmed local collision involved 35, 10, or 0.4 kV lines. Assigning 0–8 unobserved deaths among the lost birds changed overall mortality from 57.5% to 77.5% and the share represented by the 14 observed power-line deaths from 60.9% to 45.2%.

**Figure 3:**
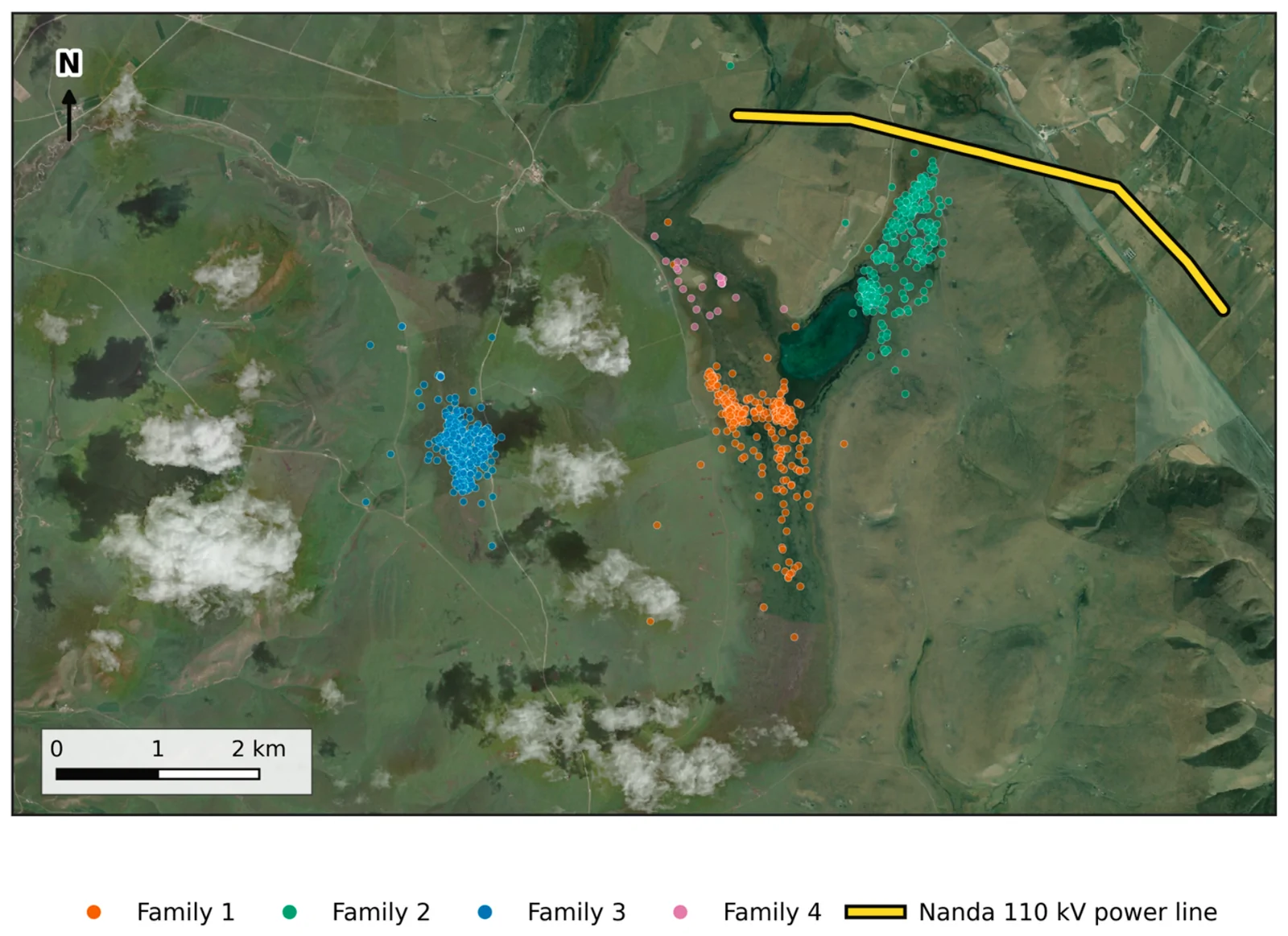
GPS-GSM locations of four fledgling Black-necked Crane families during the early pre-flight sampling period in Luanhaizi Wetland, shown as an enlarged view of the study area in Figure 1. Satellite basemap: Esri World Imagery.

**Figure 4:**
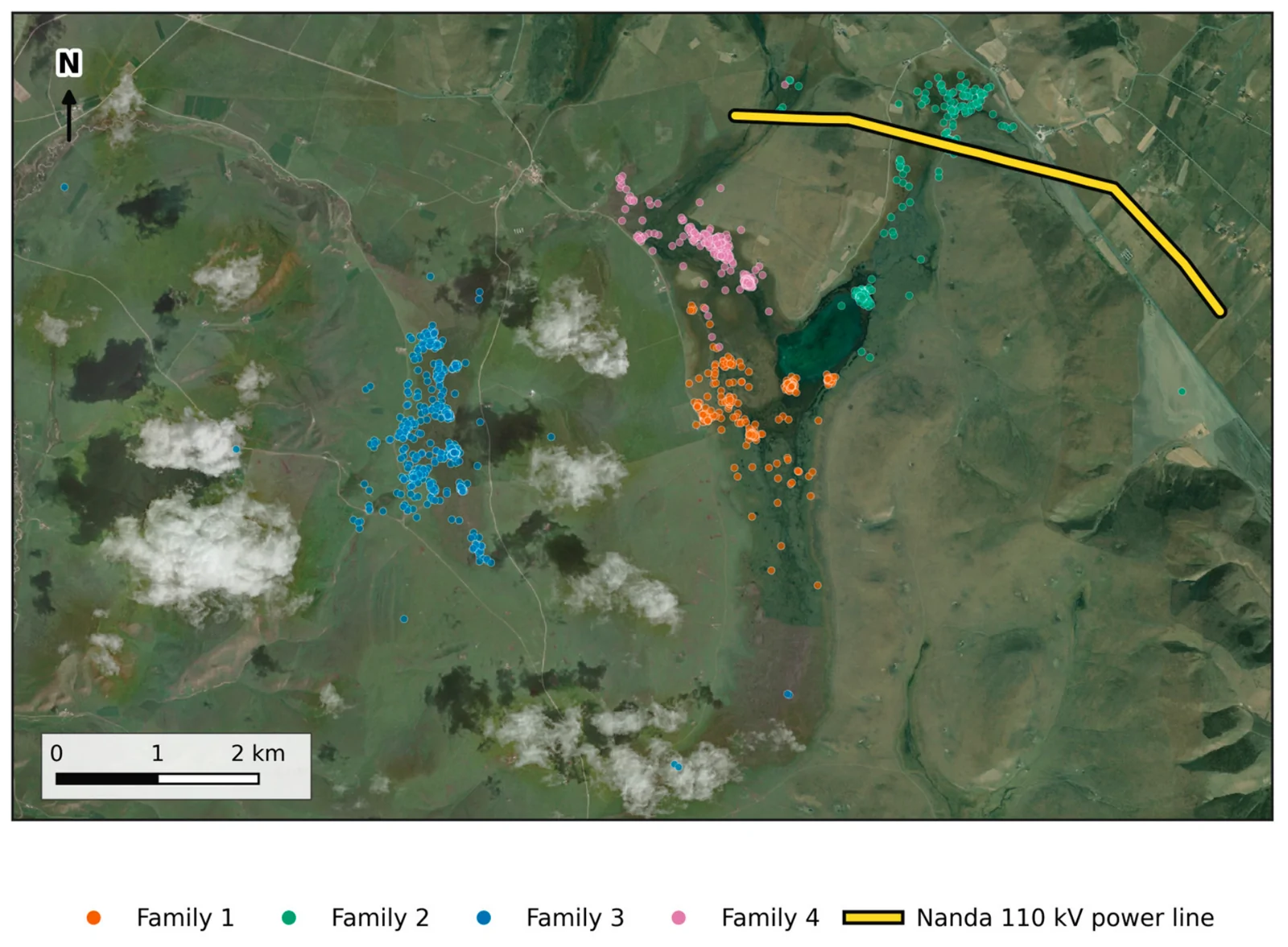
GPS-GSM locations of four fledgling Black-necked Crane families during the later flight-capable sampling period in Luanhaizi Wetland, shown as an enlarged view of the study area in Figure 1. Satellite basemap: Esri World Imagery.

Descriptive point maps from 2023 showed that recorded family locations were more spatially dispersed during the later flight-capable period than during the early pre-flight period (Figure 3 and Figure 4). Family 3 showed the greatest westward extension of recorded locations, whereas Families 1, 2, and 4 remained concentrated in the central and eastern parts of the wetland. These maps display recorded locations only; they neither quantify crossings of the 110 kV corridor nor represent formal space-use estimates or utilization distributions.

Mortality composition differed among families, but these comparisons are descriptive because family-specific exposure to fences and power lines was not quantified. Family 1 had two field-inferred power-line collision deaths, and all confirmed deaths in Family 2 were attributed to power-line collision. Most confirmed deaths in Family 3 were associated with fence entanglement, although one power-line collision also occurred. Family 4 had both fence- and collision-related deaths. Individual No. 178 illustrates that recorded spatial overlap with power lines varied over time: after its tracked movements extended towards the power-line area near Family 2, it was classified as a field-inferred 110 kV power-line collision death on 17 April 2023.

Because all 14 field-inferred power-line collision deaths involved 110 kV lines, the UAV assessment focused on one selected span within the documented 110 kV collision-associated area (Figure 2D). Standardized UAV images from this span were used to examine how environmental and acquisition conditions affected image-based line conspicuity.

### 3.2. Analysis of Power-Line Conspicuity and Image Sharpness

Across the 63 images annotated twice, the mean precision, recall, F1 score, and intersection over union (IoU) were 0.629, 0.685, 0.653, and 0.489, respectively (Table S1), indicating moderate rather than pixel-perfect spatial overlap between repeated line masks. Because the wires formed narrow linear features, small positional differences between repeated traces could substantially reduce pixel-level overlap. Nevertheless, when annotation variability was propagated through the subsequent calculations, contrast and edge strength showed good absolute agreement between annotation rounds, with ICC(A,1) values of 0.859 and 0.986, respectively. Mean VI was 17.108 ± 8.921 for the first annotations and 16.149 ± 10.567 for the second annotations. Agreement between the two VI estimates was strong (Pearson’s r = 0.901, Spearman’s ρ = 0.864, Kendall’s τ = 0.699), with ICC(A,1) = 0.885. The MAE, median absolute difference, and RMSE of VI were 3.151, 1.875, and 4.696, respectively. The mean bias of the second annotation relative to the first was −0.959, and no significant paired difference was detected (Wilcoxon signed-rank test, *p* = 0.123). The three-class assignments agreed for 50 of the 63 images (79.4%), while the Jaccard similarity of the High-concern class was 0.700. Variance decomposition attributed 88.5% of total VI variance to between-image differences and 11.5% to annotation-round and residual components. Continuous VI values and rankings were therefore more repeatable than class assignments near the data-derived boundaries.

**Figure 5:**
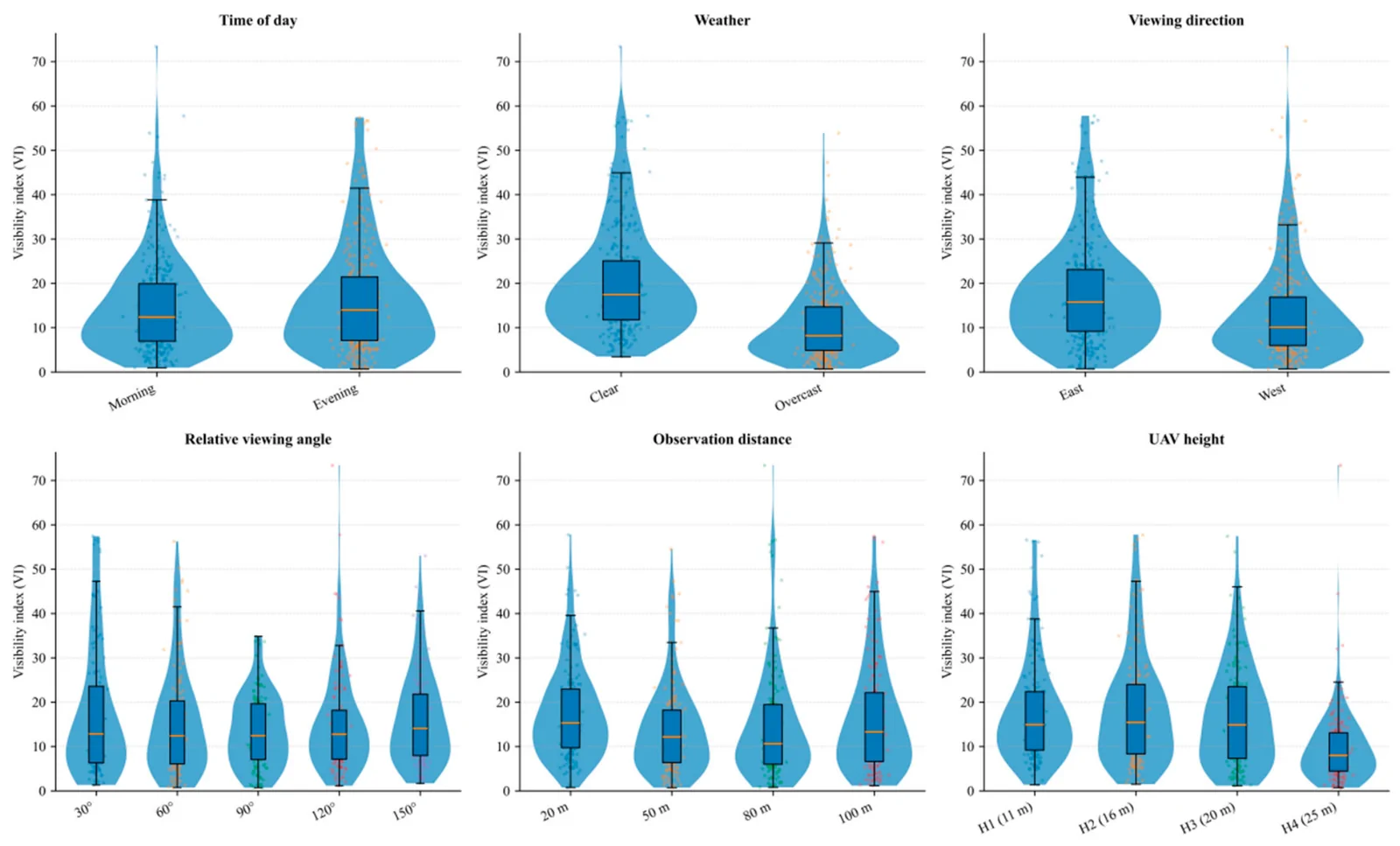
Violin plots of visibility index (VI) under different environmental factors.

VI remained stable across the tested local-background widths (Table S6). Relative to the 100 × 100-pixel reference, Spearman’s ρ was 0.926–0.985, Kendall’s τ was 0.777–0.905, and VI MAE was 0.993–2.535. Exact three-class agreement was 88.8–95.6%, and High-concern Jaccard similarity was 0.848–0.940. Agreement exceeded 95% for the adjacent 75 × 75- and 125 × 125-pixel alternatives; even the 50 × 50- and 150 × 150-pixel alternatives retained 88.8% and 90.9% agreement, respectively. Thus, the main VI ordering and concern-class patterns were not materially dependent on the 100 × 100-pixel background definition. The blocked analyses detected differences in VI between weather conditions and viewing directions, as well as among observation distances and UAV heights. VI was lower under overcast conditions than under clear conditions (Wilcoxon signed-rank test, W = 6277.000, *p* < 0.001) and also differed between viewing directions (W = 14,906.000, *p* < 0.001). Significant differences were found among observation distances (Friedman test, χ^2^ = 39.660, df = 3, *p* < 0.001) and UAV heights (χ^2^ = 116.693, df = 3, *p* < 0.001). No significant difference was detected among relative viewing angles (χ^2^ = 8.856, df = 4, *p* = 0.065), whereas VI differed between morning and evening observations (W = 22,094.000, *p* = 0.030). The complete results of the blocked tests are presented in Table S2. These standardized photographic measurements were generally highest under clear conditions, in eastward views, at shorter observation distances, and at UAV heights of 11–20 m, and lowest under overcast conditions and at 25 m (Figure 5; Table S2). These patterns should not be interpreted as direct measurements of conductor detectability by flying cranes.

**Figure 6:**
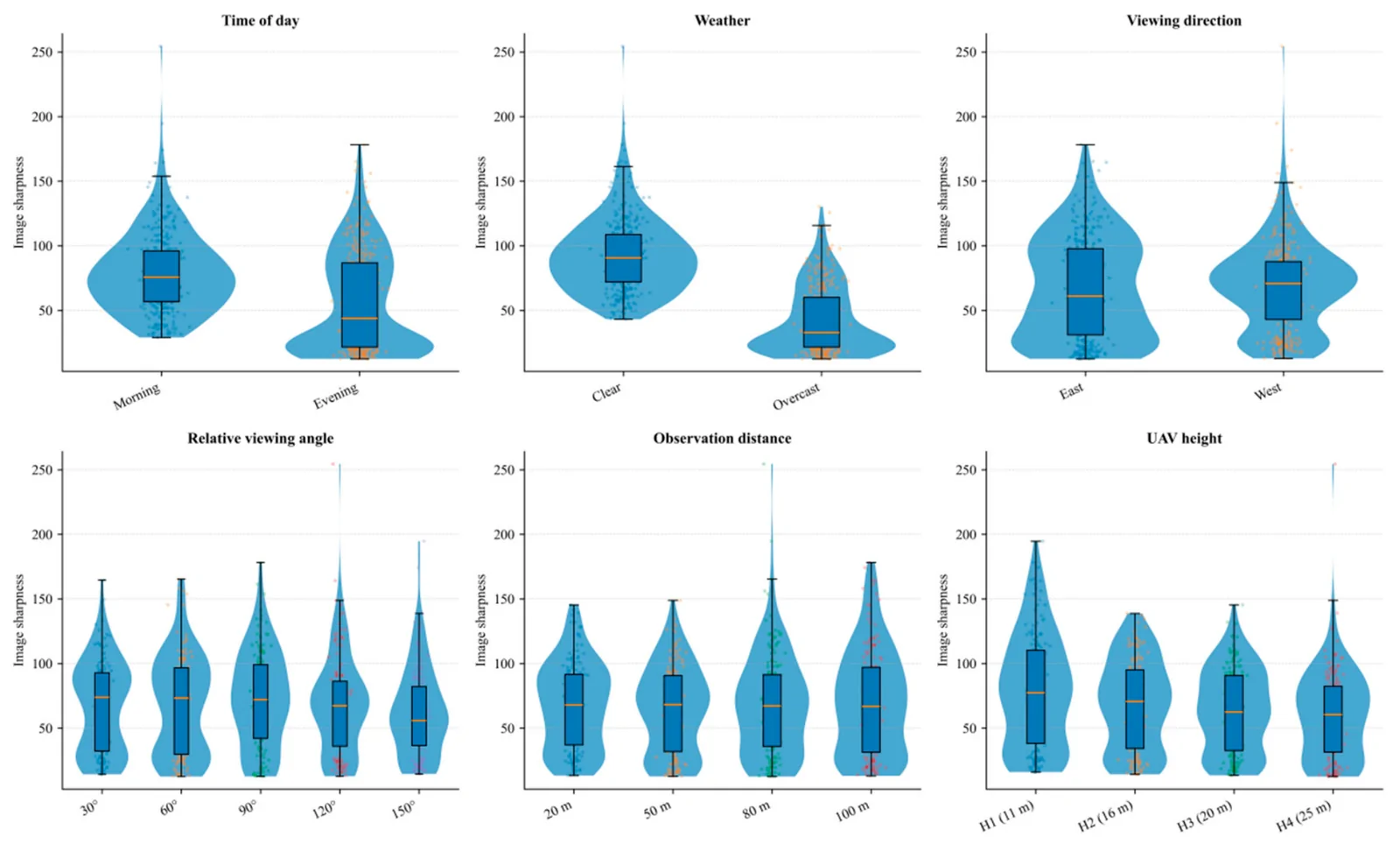
Violin plots of image sharpness under different environmental factors.

Image sharpness also differed across several experimental factors. Images obtained under clear conditions were sharper than those obtained under overcast conditions (Wilcoxon signed-rank test, W = 2454.000, *p* < 0.001), and morning images were sharper than evening images (W = 9448.000, *p* < 0.001). No significant difference in sharpness was detected between viewing directions (W = 24,243.000, *p* = 0.386). Sharpness varied significantly among relative viewing angles (Friedman test, χ^2^ = 25.919, df = 4, *p* < 0.001), observation distances (χ^2^ = 24.756, df = 3, *p* < 0.001), and UAV heights (χ^2^ = 211.552, df = 3, *p* < 0.001). These differences describe variation in image quality and were not interpreted as evidence of variation in Black-necked Crane visual perception (Figure 6).

### 3.3. Analysis of Visible Length Characteristics of Power Lines

Visible length differed across all six experimental factors. Power lines had a greater visible length under overcast conditions than under clear conditions (Wilcoxon signed-rank test, W = 18,127.000, *p* < 0.001), and visible length was greater in the morning than in the evening (W = 15,907.000, *p* < 0.001). A difference was also detected between viewing directions (W = 22,248.000, *p* = 0.038).

**Figure 7:**
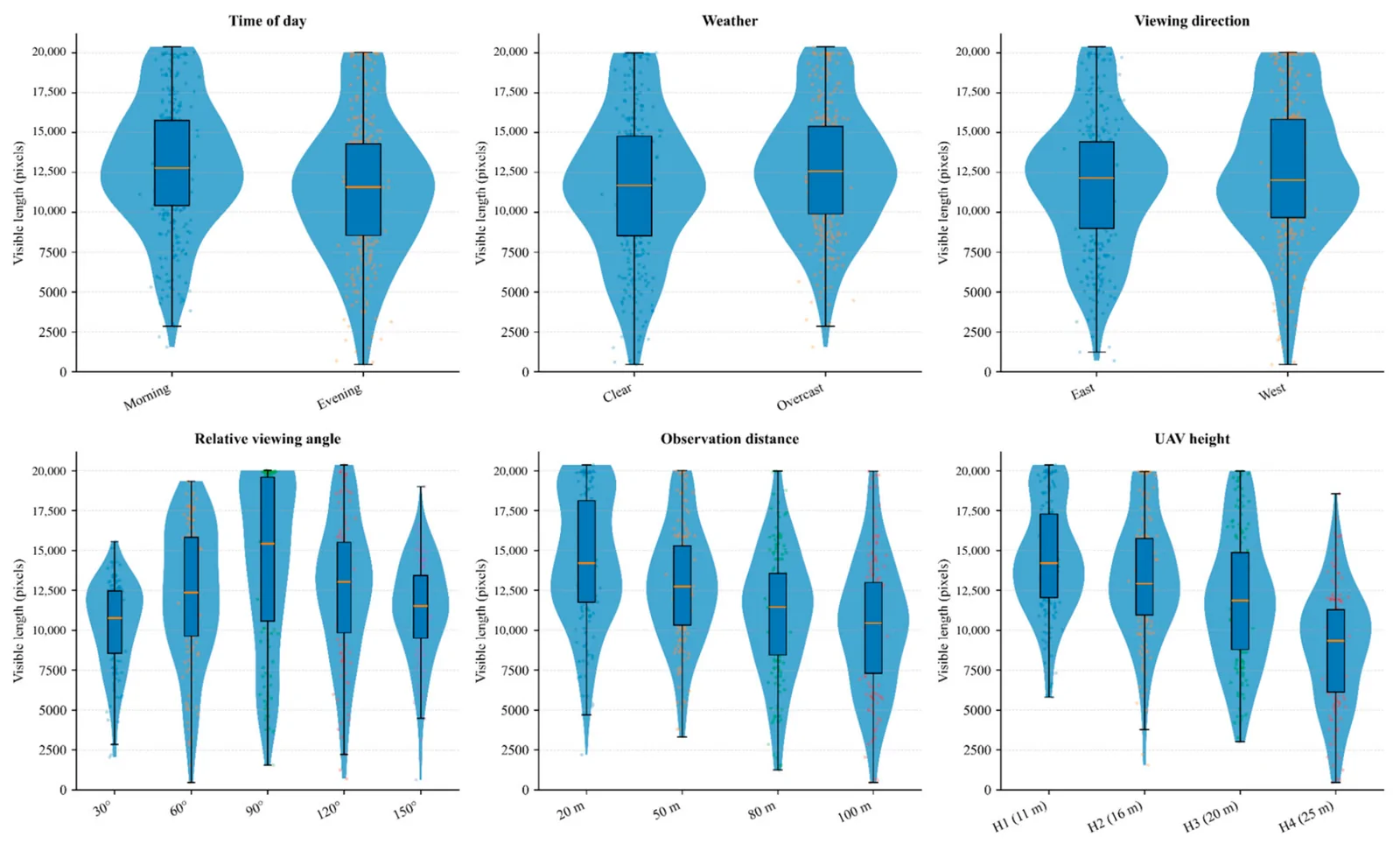
Violin plots of image-based visible length (pixels) across the six experimental factors.

The Friedman tests showed significant differences among relative viewing angles (χ^2^ = 99.000, df = 4, *p* < 0.001), observation distances (χ^2^ = 185.184, df = 3, *p* < 0.001), and UAV heights (χ^2^ = 194.205, df = 3, *p* < 0.001). Visible length was generally greater at shorter observation distances and lower UAV heights. The marked variation among viewing angles further indicated that this measure was strongly affected by acquisition geometry (Figure 7; Table S2).

Visible length represents the amount of annotated wire present in an image, but it does not describe how clearly the wire can be distinguished from its background. Its strong dependence on viewing geometry supported its use as a supplementary image variable rather than as the rimary measure of relative image-based conspicuity.

### 3.4. Descriptive VI Stratification Within the 110 kV Collision-Associated Area

Within the documented 110 kV collision-associated area, lower VI values represented lower relative image-based power-line conspicuity and, consequently, greater visibility concern within this dataset. A K-means-assisted three-level stratification was applied to the 640 observations without manually setting the two cut-points. Diagnostic results for k = 2–6 are reported in Table S3. The mean silhouette score was highest at k = 2 (0.629), compared with 0.587 at k = 3. The three-level solution was therefore not interpreted as the silhouette-optimal or naturally distinct cluster structure; it was retained to represent an intermediate screening range.

**Figure 8:**
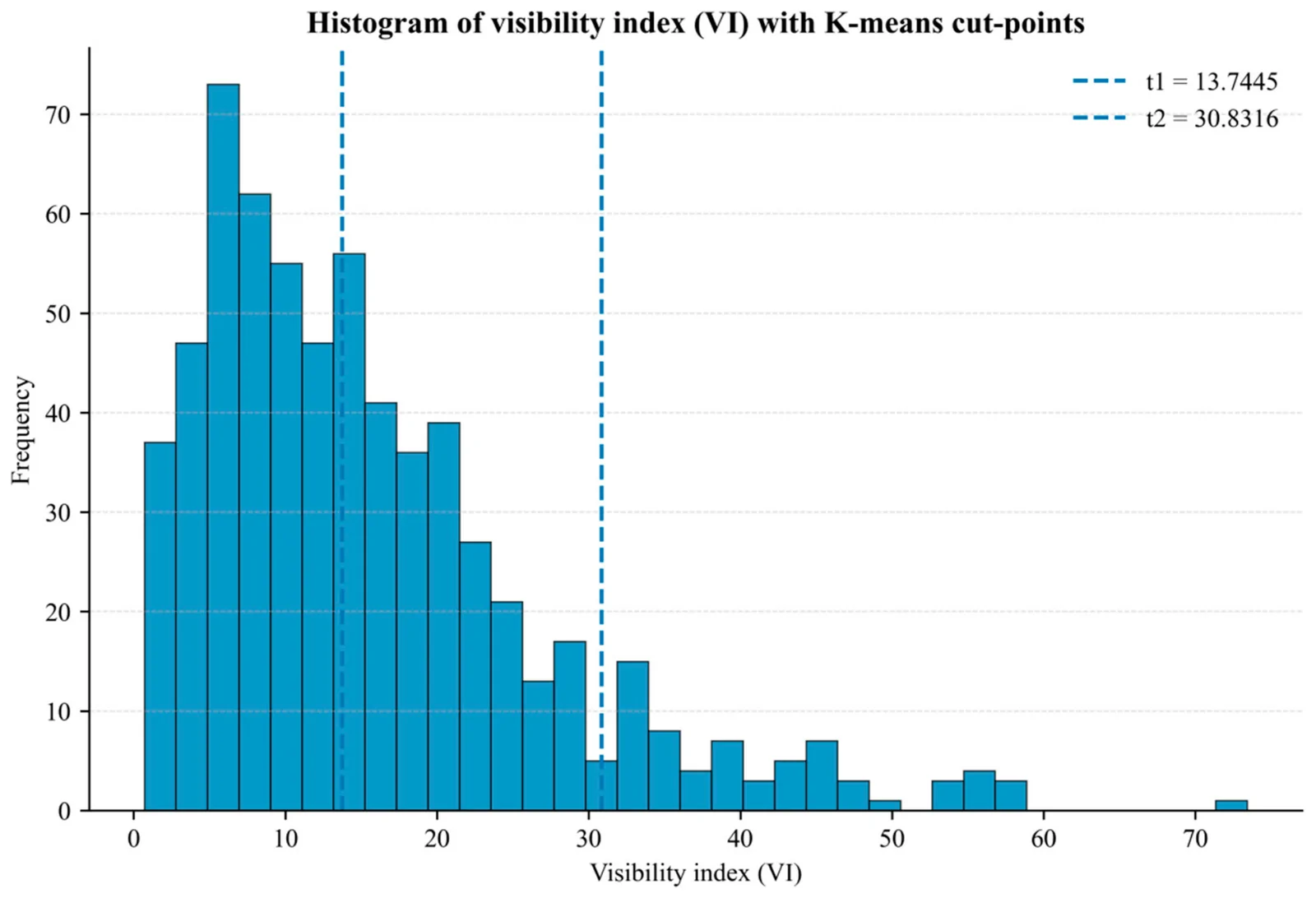
Histogram of the visibility index (VI) with data-derived cut-points for the descriptive three-level stratification. The cut-points are t_1_ = 13.7445 and t_2_ = 30.8316 (vertical dashed lines), defining High concern (VI ≤ t_1_), Medium concern (t_1_ < VI ≤ t_2_), and Low concern (VI > t_2_). The three classes contained 340, 235, and 65 observations, respectively. Lower VI indicates lower image-based line conspicuity and greater relative visibility concern within the sampled dataset.

The midpoints between adjacent centroids produced cut-points of t_1_ = 13.7445 and t_2_ = 30.8316. Observations were classified as High concern when VI ≤ 13.7445, Medium concern when 13.7445 < VI ≤ 30.8316, and Low concern when VI > 30.8316. The three classes contained 340, 235, and 65 observations, respectively (Figure 8; Table 2). These classes summarize relative image-based conspicuity within the present dataset and do not represent general collision-risk thresholds.

The VI ranking and the composition of the High-concern group were reasonably stable when the relative weights assigned to contrast and edge strength were varied. When the contrast weight was set to 0.25, Kendall’s τ and Spearman’s ρ relative to the equal-weight VI were 0.780 and 0.934, respectively. When the contrast weight was increased to 0.75, the corresponding values were 0.807 and 0.948. The High-concern groups obtained under these alternative weighting schemes had Jaccard similarities of 0.781 and 0.836, respectively, relative to the equal-weight classification (Table S4). Thus, rankings and group composition were numerically robust to the tested weights, although some observations changed; this does not establish perceptual or biological validity.

The shallow CART model was used to distinguish High-concern observations (VI ≤ 13.7445) from all other observations. Under grouped five-fold cross-validation, the mean precision, recall, and F1 score for the High-concern class were 0.682 ± 0.076, 0.744 ± 0.085, and 0.709 ± 0.064, respectively, and the overall accuracy was 0.678 ± 0.060. These values indicate moderate performance in predicting the descriptive VI class, rather than collision occurrence or mortality.

The tree associated High-concern observations mainly with overcast weather, a UAV height of 25 m, and the sampled observation-distance categories of 50, 80, and 100 m. Under overcast conditions, observations were more frequently assigned to the High-concern group when the UAV height was 25 m or the observation distance was 50, 80, or 100 m. Under clear conditions, High-concern classification occurred mainly when the 25 m UAV height was combined with one of these three distance categories. These results apply only to the discrete factor levels included in the experiment and should not be interpreted as continuous ecological thresholds.

## 4. Discussion

The mortality records and UAV analysis address related but distinct parts of this conservation problem. Tracking and field investigations identified the threat pattern and delineated the 110 kV collision-associated area. One selected span was intensively sampled across 640 factorial combinations. Holding the infrastructure constant allowed the UAV protocol to distinguish relative changes in conductor conspicuity across environmental and acquisition conditions. VI does not explain fence mortality or estimate collision probability. The experiment therefore provides a field-based proof of concept and a transferable workflow for other power-line settings, landscape backgrounds, and environmental conditions.

GPS–GSM tracking and field verification identified wire-mesh fence entanglement and power-line collision as the two field-inferred infrastructure-related mortality types documented among the monitored Black-necked Cranes. Of the 23 confirmed deaths, nine were classified as wire-mesh fence entanglements and 14 as power-line collisions; all 14 collision deaths involved 110 kV lines. The 2023 point maps showed broader spatial dispersion during the later flight-capable period, but they do not estimate utilization distributions or individual exposure. These records provide spatial context only and do not demonstrate a developmental transition in the predominant mortality source.

Mortality composition also differed among families, but these patterns should be interpreted descriptively. All confirmed deaths in Families 1 and 2 were classified as power-line collisions, whereas Family 3 had predominantly fence-related deaths and Family 4 had both mortality types. Because the lengths and densities of fences and power lines across the areas represented by each family’s GPS-GSM locations were not quantified, the observed differences cannot be used to infer family-specific exposure or vulnerability. Relative to earlier case-based accounts of Black-necked Crane collisions [27], the principal contribution of the present study is that multi-year mortality records were used to direct a line-specific UAV assessment of wire conspicuity, rather than to demonstrate a general developmental sequence in mortality.

VI varied systematically with several environmental and acquisition factors. Values were lower under overcast than clear conditions and generally decreased at greater observation distances and at the UAV height of 25 m. Differences were also detected between viewing directions and between morning and evening observations. These patterns may reflect combined variation in illumination, background, conductor orientation, camera exposure, apparent conductor width, and edge definition. Because these components were not independently measured, the effects of viewing direction and time of day cannot be assigned to a single optical mechanism.

Relative viewing angle did not significantly affect VI, although it had a marked effect on visible length. In addition, visible length was greater under overcast conditions even though VI was lower. This difference clarifies what the two variables measure. Visible length describes how much annotated wire is present in an image and is strongly influenced by acquisition geometry. A longer visible line is not necessarily more distinguishable from its background. VI instead combines target–background contrast and local edge strength and was used as the primary descriptor of relative image-based conspicuity in the present analysis.

Because normalized contrast is one of the two components of VI, its association with VI cannot be interpreted as independent validation. The alternative weighting analysis showed that image rankings and High-concern membership were reasonably stable across the tested weights, demonstrating numerical robustness within the standardized RGB dataset. It does not establish that the equal-weight VI is biologically valid or reproduces Black-necked Crane vision.

The three VI classes provide a concise, decision-oriented summary of variation within the present dataset. Although k = 2 yielded the highest silhouette score, k = 3 was retained to represent an intermediate screening range. The resulting High-, Medium-, and Low-concern classes are descriptive categories rather than evidence of three naturally distinct ecological groups. Their cut-points are specific to the sampled images; when the workflow is applied to other landscapes, seasons, or acquisition scenarios, the same three-level structure can be recalibrated using the new image dataset.

The CART model further summarized the sampled conditions associated with the descriptive High-concern VI class. Its grouped cross-validation performance was moderate, with an F1 score of 0.709 for the High-concern class and an overall accuracy of 0.678. High-concern classifications occurred mainly under overcast conditions, at the UAV height of 25 m, and at observation distances of 50, 80, or 100 m. These results identify combinations among the discrete experimental levels. They do not indicate that 25 m or any of the sampled distances constitute biological thresholds for crane collision risk.

Management in the Luanhaizi Wetland should prioritize the documented 110 kV collision-associated area, particularly portions of the line corridor used by tracked fledglings during the later flight-capable sampling period. This prioritization is based on the observed mortality history rather than a modelled estimate of collision risk. It neither demonstrates that 110 kV lines are intrinsically more dangerous than other voltage classes nor establishes that local lines without confirmed deaths are safe.

Within the documented collision-associated section, the standardized UAV protocol provides a basis for identifying candidate locations and background conditions for marker trials. It does not determine which marker design will reduce collision mortality. Candidate designs should maximize contrast with local backgrounds and may include high-contrast patterns, reflective elements, or moving components. Before wider application, their effectiveness should be evaluated using a Before–After/Control–Impact or matched-control design with standardized collision monitoring and correction for carcass persistence and searcher efficiency. Separately, the documented fence-entanglement deaths support the removal of redundant wire-mesh fences or their modification in areas used by cranes; this recommendation derives from the mortality records rather than the UAV analysis.

Mortality causes were field-inferred from concordant tracking and field evidence. Because field verification was triggered by individual tracking records rather than conducted as standardized carcass searches along all local lines, carcass persistence and searcher efficiency were not estimated. The records therefore describe detected mortality within the monitored cohort, not corrected collision frequency across the wider network. Loss to follow-up may have been non-random; the outcome-sensitivity analysis evaluated how unobserved deaths among the eight lost birds would alter the mortality summary. The spatial scope of the UAV results should be interpreted at the sampled-setting level. Intensive sampling of one selected span controlled infrastructure and acquisition conditions and enabled robust within-setting comparisons. Accordingly, location-specific VI values describe the sampled setting, while the standardized workflow can be evaluated and applied in other power-line, landscape, and acquisition scenarios. The UAV viewing geometry does not reproduce the visual perspective, movement, or decision-making of a flying crane. The images provide standardized measurements of visible-light features rather than a direct simulation of avian perception. The sampled images mainly represented conductors viewed against green terrestrial backgrounds and did not cover fog, snow, strong backlighting, or other extreme conditions. Thresholds and decision rules may change under different backgrounds or seasons.

Because only one quality-screened photograph per factorial combination was annotated, within-combination variability among the five technical repeats was not quantified. RGB imagery is device-dependent and excludes ultraviolet and receptor-specific information. Converting these camera values to CIELAB would represent human rather than crane perception; without spectral reflectance and species-specific visual parameters, an avian perceptual colour model could not be calculated. Conductor diameter was not analyzed separately, and its effect could not be isolated from viewing distance, background, or other imaging factors.

Manual tracing introduced additional uncertainty. Although repeated line masks showed only moderate pixel-level overlap, propagation of the annotation differences through the complete calculation workflow produced good absolute agreement for VI [ICC(A,1) = 0.885], strong rank consistency, and no significant paired difference between annotation rounds. The three-class assignments agreed for 79.4% of the re-annotated images, while the High-concern class had a Jaccard similarity of 0.700. These results indicate that continuous VI values and their relative ranking were more stable than the pixel-level masks themselves. Nevertheless, the less-than-complete classification agreement also shows that images close to a cluster cut-point may change categories following small annotation differences. Separately, VI rankings and concern classifications remained stable across 50 × 50- to 150 × 150-pixel dilation kernels, confirming that the 100 × 100-pixel local-background definition did not dominate the main patterns. Individual VI values and concern classes should therefore be interpreted as relative screening results rather than exact reconstructions of conductor visibility.

Further validation should test whether VI predicts collision occurrence by applying the workflow to paired collision and non-collision sections spanning different conductor arrangements, numbers of conductors, shield-wire positions, line orientations, tower spacing, seasons, and landscape backgrounds. Combining VI with GPS-derived flight trajectories and hazardous-altitude crossings could integrate standardized image conspicuity with movement-based exposure, while multispectral measurements and species-specific visual models could better approximate crane perception. Finally, post-installation matched-control monitoring is needed to test whether VI-guided marker placement reduces collision mortality.

## 5. Conclusions

Long-term GPS–GSM tracking and field verification identified wire-mesh fence entanglement and power-line collision as the two documented infrastructure-related mortality types among monitored Black-necked Cranes at the Luanhaizi Wetland. All 14 field-inferred collision deaths in this cohort involved 110 kV lines, which guided selection of a collision-associated area for standardized UAV assessment. Across 640 RGB images, the image-based VI varied systematically with weather, observation distance, viewing direction, time of day, and relative UAV height. Lower conspicuity occurred mainly under overcast conditions, at greater distances, and at the highest sampled camera position. Repeatability and sensitivity analyses showed that VI rankings were reasonably stable to annotation variation, alternative component weights, and local-background definitions.

VI is intended as a standardized measure of relative conductor conspicuity in RGB images, rather than a direct estimate of crane visual perception or collision probability. Nevertheless, the combined workflow provides a reproducible way to use documented mortality to identify candidate areas, compare photographic conditions, and design targeted mitigation trials. When applied to other power-line, landscape, seasonal, or acquisition scenarios, the workflow can be recalibrated with local imagery and paired with standardized collision monitoring to test whether marker placement reduces mortality. In parallel, removal or modification of redundant wire-mesh fences can address the complementary mortality evidence documented in the monitored cohort.

## Figures and Tables

**Table 1:** Monitoring outcomes for 40 Black-necked Cranes at Luanhaizi Wetland, 2020–2025.

ID	Family	Tracking Date	Monitoring Status	Field-Inferred Cause of Death	Outcome Date
111	1	30 July 2021	Survival		
112	1	30 July 2021	Survival		
178	1	27 July 2022	Death	Power line	17 April 2023
217	1	15 August 2023	Survival		
229	1	29 July 2024	Lost to follow-up		21 September 2024
246	1	17 August 2025	Death	Power line	18 October 2025
247	1	17 August 2025	Survival		
58	2	27 July 2020	Survival		
59	2	27 July 2020	Death	Power line	28 September 2020
117	2	31 July 2021	Death	Power line	5 May 2022
118	2	31 July 2021	Lost to follow-up		25 November 2021
167	2	24 July 2022	Death	Power line	3 October 2022
168	2	24 July 2022	Death	Power line	26 May 2024
215	2	15 August 2023	Death	Power line	8 May 2025
216	2	15 August 2023	Death	Power line	17 October 2023
242	2	16 August 2025	Survival		
GS	2 Father		Death	Power line	15 October 2025
56	3	26 July 2020	Death	Fence	3 August 2020
57	3	26 July 2020	Death	Fence	31 July 2020
113	3	30 July 2021	Survival		
114	3	30 July 2021	Death	Fence	3 October 2021
169	3	7 July 2022	Lost to follow-up		26 September 2022
170	3	7 July 2022	Lost to follow-up		27 September 2022
220	3	16 August 2023	Lost to follow-up		4 June 2025
221	3	15 August 2023	Lost to follow-up		21 November 2023
230	3	9 August 2024	Death	Fence	24 August 2024
231	3	9 August 2024	Death	Fence	24 August 2024
243	3	16 August 2025	Death	Power line	28 October 2025
54	4	26 July 2020	Death	Fence	14 September 2020
115	4	31 July 2021	Death	Power line	4 October 2021
116	4	31 July 2021	Death	Fence	3 September 2021
176	4	27 July 2022	Lost to follow-up		17 May 2024
177	4	27 July 2022	Death	Power line	8 October 2022
218	4	15 August 2023	Death	Power line	19 June 2024
219	4	15 August 2023	Death	Fence	6 October 2023
232	4	9 August 2024	Lost to follow-up		27 September 2024
233	4	9 August 2024	Survival		
244	4	16 August 2025	Death	Fence	16 September 2025
245	4	16 August 2025	Survival		
179	Adult	24 July 2022	Death	Power line	14 July 2024

**Table 2:** Descriptive three-level grouping of VI values and group sizes.

Class	Definition	n (%, N = 640)
High	VI ≤ 13.7445	340 (53.1%)
Medium	13.7445 < VI ≤ 30.8316	235 (36.7%)
Low	VI > 30.8316	65 (10.2%)

## Data Availability

Image-level acquisition metadata and derived metrics for the 640 analyzed photographs are provided as Data S1-analysis_results, and the analysis code is provided as Code S1. Summary results are included in the article and its Supplementary Materials. The complete anonymized UAV image set and corresponding annotation masks are available from the corresponding author upon reasonable request, subject to applicable data-use restrictions. Precise GPS-GSM locations and unredacted image metadata are not publicly available because their disclosure could reveal sensitive locations of a Class I nationally protected species and detailed infrastructure locations.

## Supplementary Materials

The following supporting information can be downloaded at: https://www.mdpi.com/article/10.3390/ani16172698/s1, Table S1, Repeatability of manual power-line delineation and propagation of annotation variability to derived image metrics and VI; Table S2, Summary of blocked non-parametric tests for environmental effects on VI; Table S3, Diagnostic statistics for candidate one-dimensional K-means solutions (k = 2–6); Table S4, Sensitivity of VI ranking and High-concern classification to alternative contrast and edge-strength weights; Table S5, Cause-specific mortality incidence rates among tagged Black-necked Cranes; Table S6, Sensitivity of VI rankings and concern classifications to alternative local-background dilation-kernel sizes; Data S1-analysis_results, Image-level acquisition metadata and derived metrics for the 640 analyzed photographs; Code S1, Python scripts used for image processing, VI calculation, statistical analysis, and validation.
