# Supplementary material for: Mortality Records Guide UAV-Based Assessment of Power-Line Conspicuity at a Black-Necked Crane Breeding Site
Source: Animals (Basel). 2026 Aug 31;16(17):2698. doi: 10.3390/ani16172698 (PMC13564337; doi:10.3390/ani16172698)
Supplement: Supplementary file 1 [file animals-16-02698-s001.zip › Tables S1-S6.pdf]

Table S1. Repeatability of manual power-line delineation and propagation of annotation variability to derived image metrics and VI.

**Outcome**	**Agreement measure**	**Result**
Contrast	Pearson's *r*	0.878
Contrast	Spearman's ρ	0.831
Contrast	ICC(A,1)	0.859
Contrast	MAE	2.899
Edge strength	Pearson's *r*	0.988
Edge strength	Spearman's ρ	0.989
Edge strength	ICC(A,1)	0.986
Edge strength	MAE	468,549.528
VI	First annotation, mean ± SD	17.108 ± 8.921
VI	Second annotation, mean ± SD	16.149 ± 10.567
VI	Pearson's *r*	0.901
VI	Spearman's ρ	0.864
VI	Kendall's τ	0.699
VI	ICC(A,1)	0.885
VI	MAE	3.151
VI	Median absolute difference	1.875
VI	RMSE	4.696
VI	Mean bias, second − first	−0.959
VI	95% limits of agreement	−10.041 to 8.124
VI	Paired Wilcoxon *p*	0.123
Three-class assignment	Exact agreement	50/63（79.4%）
High-concern class	First/second annotation	22/29
High-concern class	Jaccard similarity	0.700

Table S2. Summary of blocked non-parametric tests for environmental effects on VI.

**Response**	**Factor**	**Test**	**Blocks**	**Statistic**	**p-value**	**Interpretation**
VI	Weather	Wilcoxon signed-rank	320	W = 6277.000	$p < 0.001$	significant
VI	Direction	Wilcoxon signed-rank	320	W = 14906.000	$p < 0.001$	significant
VI	Time of day	Wilcoxon signed-rank	320	W = 22094.000	$p = 0.030$	weak effect
VI	Viewing	Friedman	128	$\chi^2 = 8.856$	p =	not significant

Response	Factor	Test	Blocks	Statistic	p-value	Interpretation
	angle				0.065	
VI	Distance	Friedman	160	$\chi^2 = 39.660$	$p < 0.001$	significant
VI	Height	Friedman	160	$\chi^2 = 116.693$	$p < 0.001$	significant

Table S3. Diagnostic statistics for candidate one-dimensional K-means solutions (k = 2 – 6).

k	WCSS / Inertia	Average silhouette score	Class sizes
2	30321.273	0.629	493 / 147
3	13927.153	0.587	340 / 235 / 65
4	8173.685	0.576	274 / 221 / 108 / 37
5	5434.387	0.555	219 / 194 / 137 / 61 / 29
6	4036.186	0.539	158 / 156 / 139 / 110 / 50 / 27

Table S4. Sensitivity of VI ranking and High-concern classification to alternative contrast and edge-strength weights.

Contrast weight	Edge-strength weight	Kendall's τ	Spearman's ρ	High-concern Jaccard similarity
0.25	0.75	0.780	0.934	0.781
0.50	0.50	1.000	1.000	1.000
0.75	0.25	0.807	0.948	0.836

Table S5. Cause-specific mortality incidence rates among tagged Black-necked Cranes.

Mortality outcome	Person-time (bird-years)	Events	Incidence per bird-year	Exact 95% CI
All confirmed deaths	37.66	22	0.584	0.366–0.884
Power-line collision	37.66	13	0.345	0.184–0.590
Fence entanglement	37.66	9	0.239	0.109–0.454

Table S6: Sensitivity of VI rankings and concern classifications to alternative local-background dilation-kernel sizes.

Elliptical dilation kernel (pixels)	n	Spearman's ρ	Kendall's τ	VI MAE	Exact class agreement, n (%)	High-concern Jaccard
50 × 50	640	0.926	0.777	2.535	568 (88.8%)	0.848

Elliptical dilation kernel (pixels)	n	Spearman's ρ	Kendall's τ	VI MAE	Exact class agreement, n (%)	High-concern Jaccard
75 × 75	640	0.981	0.889	1.233	609 (95.2%)	0.932
100 × 100	640	1.000	1.000	0.000	640 (100.0%)	1.000
125 × 125	640	0.985	0.905	0.993	612 (95.6%)	0.940
150 × 150	640	0.943	0.815	1.919	582 (90.9%)	0.883
